# Skipping breakfast and its association with sociodemographic characteristics, night eating syndrome, and sleep quality among university students in Bangladesh

**DOI:** 10.1186/s40795-024-00860-y

**Published:** 2024-03-04

**Authors:** Md Shafiqul Islam Khan, Trisha Paul, Md. Hasan Al Banna, Mohammad Hamiduzzaman, Cornelius Tengan, Bernard Kissi-Abrokwah, Justice Kanor Tetteh, Faria Hossain, Md. Shajadul Islam, Keith Brazendale

**Affiliations:** 1 Department of Food Microbiology, Faculty of Nutrition and Food Science, Patuakhali Science and Technology University, 8602 Patuakhali, Bangladesh; 2 Faculty of Nutrition and Food Science, Patuakhali Science and Technology University, 8602 Patuakhali, Bangladesh; 3 Nutrition Initiative, Kushtia, Bangladesh; 4 School of Health Sciences, The University of Sydney, Sydney, Australia; 5 Department of Hospitality, Catering and Institutional Management, Bolgatanga Technical University, Sumbrugu, Ghana; 6 C. K. Tedam University of Technology and Applied Sciences, Navrongo, Ghana; 7 Department of Population and Health, University of Cape Coast, Cape Coast, Ghana; 8 Department of Health Sciences, University of Central Florida, Orlando, FL USA

**Keywords:** Breakfast skipping, Prevalence, Factors, University students, Bangladesh

## Abstract

**Introduction:**

Skipping breakfast has become more common, and it can significantly affect a person’s health, performance, mood, and other physiological and psychological factors. In Bangladesh, university students often encounter unhealthy dietary habits, which raises questions about why many university students choose to skip breakfast. The purpose of this study was to investigate the prevalence of skipping breakfast among university students in Bangladesh and explore the contributing factors.

**Methods:**

Patuakhali Science and Technology University, Bangladesh was the location of this cross-sectional study. Breakfast consumption was measured with the single-question item, “How often do you eat breakfast?” (Almost every day, sometimes, rarely, or never). Skipping breakfast was classified as respondents selecting sometimes, rarely, or never having breakfast. Sociodemographic, behavioral, and sleep-related data were collected as key predictor variables. Multiple logistic regression models identified factors associated with skipping breakfast.

**Results:**

The prevalence of skipping breakfast among study participants (*N* = 502, 51.6% female and mean age 21.31 years) was 63.5%. Female students were more likely to skip breakfast compared to male students (adjusted odds ratio, AOR = 1.65, 95% CI: 1.06–2.55). Smoker participants had a higher likelihood of skipping breakfast compared to non-smokers (AOR = 3.92, 95% CI: 1.57–9.78). Students with night eating syndrome had a higher likelihood of skipping breakfast compared to their counterparts (AOR = 1.84, 95% CI: 1.06–3.22). Students with poor sleep quality were three times more likely to skip breakfast than their counterparts (AOR = 2.95, 95% CI: 1.93–4.51). Overweight/obese students were less likely to skip breakfast compared to their counterparts (AOR = 0.40, 95% CI: 0.20–0.82).

**Conclusion:**

This study highlights a high prevalence of skipping breakfast among university students in Bangladesh. Specifically, students who are females, smokers, poor sleepers and who have night eating syndrome are more likely to skip breakfast compared to their counterparts. These findings underscore a need for targeted interventions and educational programs to promote healthy breakfast habits. Addressing these modifiable risk factors can have a positive impact on students’ nutritional practices and their health and wellbeing.

## Introduction

Breakfast is the first meal of the day occurring in the morning after ‘breaking’ the fast that has just occurred, typically the preceding segment of time spent sleeping. Breakfast can influence the circadian rhythm of the human body and impact physical, mental, and behavioral changes in a 24-hour cycle of the human biological system [1]. Breakfast in the right proportion and quality can regulate the body’s intake of nutrients such as carbohydrates, calories, dietary fiber, and micronutrients [2]. Other benefits associated with regular breakfast consumption include enhanced memory recall, mood, intellectual performance, and energy levels, and a reduced risk of type 2 diabetes [3–5].

Despite the numerous benefits associated with daily breakfast consumption, the number of people who ‘skip’ breakfast– hereon defined as skipping breakfast - is globally estimated to be 10 to 30% among children and adolescents [6] and 48% among university students (median age 20 years and 28 countries in Africa, the Americans and Asia) [2]. Skipping breakfast is defined as missing or foregoing breakfast at least once a week either deliberately or unintentionally [7–9]. Reported health consequences of skipping breakfast include an increased risk of overweight, type 2 diabetes, hypoglycemia, hair loss, and migraine headaches [10, 11].

In Bangladesh, skipping breakfast is a common practice. Studies conducted on university students and adults in Bangladesh have reported the prevalence of skipping breakfast can range from approximately 35 to 65% [1, 12, 13]. Less is known about factors associated with skipping breakfast among university students. Potential predictors of interest may be sleep quality and eating disorders, such as night eating syndrome. Whereas sleep quality refers to an individual’s overall satisfaction of an entire sleep experience including the duration, latency, efficiency and wake after sleep onset [14]. Night eating syndrome refers to the urge, habit, or pattern of eating late at night after dinner [15]. Night eating syndrome in university students may be worth exploring as students are more likely to be awake later at night due to academic (e.g., studying) or social (e.g., going out with friends) activities. Thus, sleep quality and night eating syndrome may be a reason for the high prevalence of skipping breakfast in university students (55–65%) [1, 2, 13].

A prior multi-country study among university students reported a higher likelihood of infrequent and/or frequent breakfast skipping among participants with sleep issues like short sleep, long sleep, sleep problems, and restless sleep [2]. Another study in Japanese women has noted a positive association between night eating syndrome (late dinners or bedtime snacks) and skipping breakfast [16]. Yet, there is a lack of evidence in Bangladesh, especially among university students. This is of particular importance as they are a population that has been characterized by a high prevalence of skipping breakfast [1, 13], poor sleep quality [17], and eating disorders [18]. Given the adverse health outcomes related to skipping breakfast, additional factors, such as sociodemographic parameters, sleep quality, and night eating syndrome, warrant investigation among university students in Bangladesh to identify those most at risk [1]. Thus, this study sets out to explore the association between sociodemographic factors, night eating syndrome, sleep quality and skipping breakfast among university students in Bangladesh. Empirical information on the prevalence of factors associated with skipping breakfast in Bangladeshi university students could inform public health programs and policies targeted at improving the overall health of university students in Bangladesh.

## Methods and materials

### Study setting

The current study was performed at Patuakhali Science and Technology University (PSTU), which is the first public institution in the Barishal division of Bangladesh and covers an area of 89.97 acres. This university has two campuses: (i) the main campus which is located in the Dumki sub-district under the Patuakhali district of Bangladesh; and (ii) the outer campus (Barishal, Babugong). Specifically, this study was based on the main campus of this university, which is positioned around 20 km north of the district center of Patuakhali and 38 km south of Barishal city. This institution provides full residential facilities to all students considering the geographical location is in a more rural location. The main campus has six residential halls (i.e., dormitories), of which four are allocated for male students and two for female students. Approximately 3,000 students are enrolled across eight faculties on the main campus of this university.

### Study design and participants

This cross-sectional study was conducted among university students from March to June 2023. Participants were considered eligible for this study if the following criteria were met: (i) undergraduate student, (ii) ≥ 18 years old, (iii) residing in the university halls, and (iv) being Bangladeshi citizens. Graduate students and those with serious medical complications or psychiatric problems were excluded from this study.

A simple random sampling technique was applied to select study participants. Trained data collectors, who were university students, went door-to-door in each of the six dormitories after getting the appropriate permission from the university authorities. Public university halls in Bangladesh do not allow females to enter male halls and vice-versa, male data collectors visited male halls and female data collectors visited female halls to collect consent and survey data. Upon permission from the students, data collectors visited the rooms explained the study objectives to the participants and assessed for study eligibility criteria. Each room had an average of three students (around 3 to 4 students per room) and one participant was selected at random. The names of the room members who were present during the survey were written on different pieces of paper, folded, shuffled, and then one paper was chosen to recruit study participants. If only a single member was present in the room during the survey, the room was excluded. Moreover, data collectors randomly approached students at the places where they gather and enjoy leisure time like playgrounds, canteen, etc. to participate in this study.

### Sample size calculation

The sample size was calculated using a single population proportion test by considering the following assumptions: (i) a 53.85% prevalence of skipping breakfast among Bangladeshi public university students was used (*p* = 0.54) [1], (ii) 95% confidence level (Z = 1.96), and (iii) 5% margin of error (d = 0.05). The calculation formula is as follows:

Minimum sample size,

$$\left[ {n=\frac{{{z^2} \times p \times (1 - p)}}{{{d^2}}}=\frac{{{{(1.96)}^2} \times 0.54 \times (1 - 0.54)}}{{{0.05^2}}}}\right]=381.7\approx382$$

A 10% non-response rate was also considered, resulting in an optimal sample size of 420 (382 + 38). A total sample of 502 participants was recruited this study.

### Description of the study variables

**Fig. 1:**
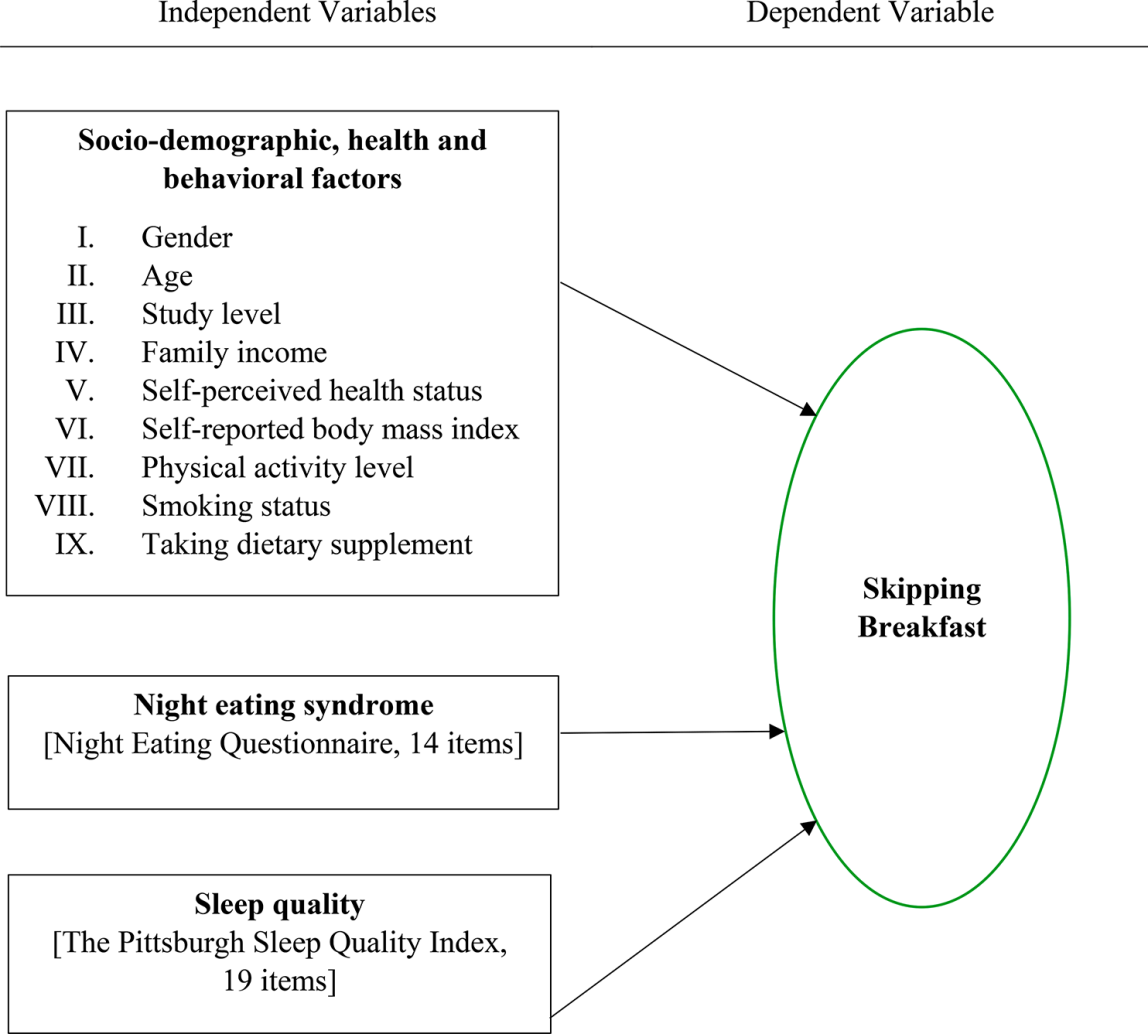
Conceptual framework of potential predictors of skipping breakfast in Bangladeshi university students

There was a total of 12 variables in this study, with 11 independent variables and one dependent variable (see Fig. 1).

Skipping breakfast was the dependent variable of this study. Breakfast consumption was assessed with the item, “How often do you eat breakfast?” There were four possible options for this question such as almost every day, sometimes, rarely, or never [2, 19]. In other studies, skipping breakfast was defined as responses marked as sometimes, rarely, or never having breakfast [2].

Participants’ sociodemographic, health, and behavioral characteristics such as age, gender (male vs. female), academic standing (1st, 2nd, 3rd or 4th year of university at undergraduate level), monthly family income, Self-perceived health status (very good, good, fair or poor), self-reported body mass index (underweight, normal weight or overweight/obese), physical activity level (inactive, moderate activity or regular activity), smoking status (yes vs. no) and the use of dietary supplements (yes vs. no) were obtained as covariates.

Participants’ Night Eating Syndrome (NES) was assessed by a validated Night Eating Questionnaire (NEQ) [20]. The NEQ evaluates the behavioral and psychological symptoms of NES. The NEQ consists of 14 questions with a 5-point Likert scale (score 0 to 4). All items, except for item number 13, were totaled up to create a total score, which ranges from 0 to 52. Generally, the NEQ employs a clinical cut-off score of ≥ 25 for a broad assessment and 30 for a higher level of specificity [21]. For this study, a clinical cut-off score (≥ 25) was used as this was a screening study.

We used a 19-item Pittsburgh Sleep Quality Index (PSQI) to assess participants’ sleep quality over the past month [22]. The PSQI is subdivided into seven components and each component could obtain a score between 0 and 3. An individual’s seven component scores, which range from 0 to 21, are summed to yield a global PSQI score for sleep quality. A score of > 5 on the global PSQI indicates poor sleep quality. Previous epidemiological investigations used the PSQI as a sleep quality screener among a variety of subpopulations, including Bangladeshi students [17].

### Study protocol

The study protocol was evaluated and approved by the Institutional Ethical Committee (IEC) of Patuakhali Science and Technology University, Bangladesh [PSTU/IEC/2023/61]. All study procedures were carried out in accordance with relevant guidelines and regulations. Written informed consent was obtained from all participants after explaining the objectives of the study. Participation was voluntary and personal data were kept confidential. Survey data were collected through face-to-face interviews using a structured questionnaire containing the various measures outlined above. The questionnaire was prepared by reviewing several relevant studies [2, 17, 20, 22]. All study team members participated in a data collection training led by the principal investigator of this study who provided instructions on the sampling approach, the consent process, the eligibility requirements, and the completion of the survey(s). The draft version of the questionnaire was piloted among a random group of students (*n* = 15) to identify any confusing or unclear questions and to acquire a better perspective on the length of time required for the interview. The data of the pilot survey were not included in the present study’s results. The survey took approximately 12–18 min to complete.

### Data analysis

Data were analyzed by Statistical Package for the Social Sciences (SPSS) software (SPSS, IBM version 23.0, Armonk, NY, USA). Descriptive statistics such as frequencies, percentages, and means were computed to summarize the variables of interest. A chi-square test assessed the distribution of skipping breakfast across predictor variables. A binary logistic regression analysis was performed to identify the factors associated with skipping breakfast. The regression models’ fitness criteria were checked by the Hosmer and Lemeshow goodness of fit test. The odds ratio with a 95% confidence interval (CI) was estimated for both unadjusted and adjusted regression models. An odds ratio plot was constructed for visual display of the adjusted regression model, and p-value < 0.05 was considered statistically significant.

## Results

### Sample characteristics

A total of 502 students participated in this study. The mean age of participants was 21.31 (SD: 1.19) years. Nearly half (51.6%) of the participants were female. Approximately 19.5% of the participants had a night-eating syndrome. More than two-thirds of the participants showed poor sleep quality (68.3%) (Table 1).

**Table 1:** Socio-demographic, health, and behavioral characteristics of study participants (*n* = 502)

Variable(s)	Frequency	Percentage (%)
**Sex**		
Male	243	48.4
Female	259	51.6
**Age (in years)**		
18–21	297	59.2
22–26	205	40.8
**Study level**		
1st year	322	64.1
2nd year	92	18.3
3rd year	50	10.0
4th year	38	7.6
**Monthly family income (BDT)**		
<20,000	47	9.4
20,000–40,000	254	50.6
>40,000	201	40.0
**Self-perceived health status**		
Very good	49	9.8
Good	314	62.5
Fair	113	22.5
Poor	26	5.2
**Self-reported body mass index (BMI)**		
Underweight	86	17.1
Normal weight	372	74.1
Overweight/obese	44	8.8
**Physical activity level**		
Physically inactive	59	11.8
Moderate activity	257	51.2
Regular activity	186	37.1
**Smoking status**		
Yes	47	9.4
No	455	90.6
**Taking dietary supplements**		
Yes	72	14.3
No	430	85.7
**Night eating syndrome**		
Yes	98	19.5
No	404	80.5
**Overall sleep quality**		
Good	159	31.7
Poor	343	68.3

### Prevalence and factors associated with skipping breakfast

The prevalence of skipping breakfast among study participants was 63.5% (*n* = 319). The chi-square test found that participants’ self-perceived health status (*p* = 0.009), smoking status (*p* = 0.004), taking dietary supplements (*p* = 0.014), night eating syndrome (*p* = 0.006), and sleep quality (*p* < 0.001) were significantly associated with breakfast skipping (Table 2).

**Table 2:** Presentation of skipping breakfast by participants’ characteristics (*N* = 502)

Variable(s)	Skipping Breakfast; n (%)		P value
	Yes	No	
Gender			0.315
Male	149 (61.3)	94 (38.7)	
Female	170 (65.6)	89 (34.4)	
Age (in years)			0.421
18–21	193 (65.0)	104 (35.0)	
22–26	126 (61.5)	79 (38.5)	
Study level			0.329
1st year	211 (65.5)	111 (34.5)	
2nd year	58 (63.0)	34 (37.0)	
3rd year	26 (52.0)	24 (48.0)	
4th year	24 (63.2)	14 (6.8)	
Monthly family income (BDT)			0.731
<20,000	85 (66.4)	43 (33.6)	
20,000–40,000	179 (62.4)	108 (37.6)	
>40,000	55 (63.2)	32 (36.8)	
Self-perceived health status			**0.009**
Very good	35 (71.4)	14 (28.6)	
Good	182 (58.0)	132 (42.0)	
Fair	84 (74.3)	29 (25.7)	
Poor	18 (69.2)	8 (30.8)	
Self-reported body mass index			0.139
Underweight	51 (59.3)	35 (40.7)	
Normal weight	245 (65.9)	127 (34.1)	
Overweight/obese	23 952.3)	21 (47.7)	
Physical activity level			0.614
Physically inactive	39 (66.1)	20 (33.9)	
Moderate activity	158 (61.5)	99 (38.5)	
Regular activity	122 (65.6)	64 (34.4)	
Smoking status			**0.004**
Yes	39 (83.0)	8 (17.0)	
No	280 (61.5)	175 (38.5)	
Taking dietary supplements			**0.014**
Yes	55 (76.4)	17 (23.6)	
No	264 (61.4)	166 (38.6)	
Night eating syndrome			**0.006**
Yes	74 (75.5)	24 (24.5)	
No	245 (60.6)	159 (39.4)	
Overall sleep quality			**< 0.001**
Good	76 (47.8)	83 (52.2)	
Poor	243 (70.8)	100 (29.2)	

*Note* Bolded values indicate statistically significant (*p* < 0.05)

In Table 3, unadjusted binary logistic regression demonstrates the factors associated with skipping breakfast among study participants. The following factors were found to be associated with skipping breakfast: (i) being smoker (crude odds ratio, COR = 3.05, 95% CI: 1.39 to 6.67), (ii) taking dietary supplements (COR = 2.03, 95% CI: 1.14 to 3.62), (iii) having night eating syndrome (COR = 2.00, 95% CI: 1.21 to 3.31), and (iv) poor sleep quality (COR = 2.65, 95% CI: 1.79 to 3.91).

**Table 3:** Unadjusted binary logistic regression showing the factors associated with skipping breakfast among study participants

Variable(s)	Unadjusted Regression Model			
	Odds ratio	95% confidence interval		P value
		Lower limit	Upper limit	
Gender				
Male	Reference			
Female	1.21	0.84	1.73	0.315
Age (in years)				
18–21	1.16	0.80	1.68	0.421
22–26	Reference			
Study level				
1st year	1.11	0.55	2.23	0.772
2nd year	0.99	0.45	1.18	0.990
3rd year	0.63	0.27	1.49	0.296
4th year	Reference			
Monthly family income (BDT)				
<20,000	Reference			
20,000–40,000	0.72	0.37	1.42	0.345
>40,000	0.71	0.36	1.42	0.334
Self-perceived health status				
Very good	1.11	0.39	3.13	0.842
Good	0.61	0.26	1.45	0.266
Fair	1.29	0.51	3.27	0.596
Poor	Reference			
Self-reported body mass index (BMI)				
Underweight	0.76	0.47	1.22	0.253
Normal weight	Reference			
Overweight/obese	0.57	0.30	1.07	0.078
Physical activity level				
Physically inactive	Reference			
Moderate activity	0.82	0.45	1.48	0.509
Regular activity	0.98	0.53	1.81	0.943
Smoking status				
Yes	3.05	1.39	6.67	**0.005**
No	Reference			
Taking dietary supplements				
Yes	2.03	1.14	3.62	**0.016**
No	Reference			
Night eating syndrome				
Yes	2.00	1.21	3.31	**0.007**
No	Reference			
Overall sleep quality				
Good	Reference			
Poor	2.65	1.79	3.91	**< 0.001**

**Fig. 2:**
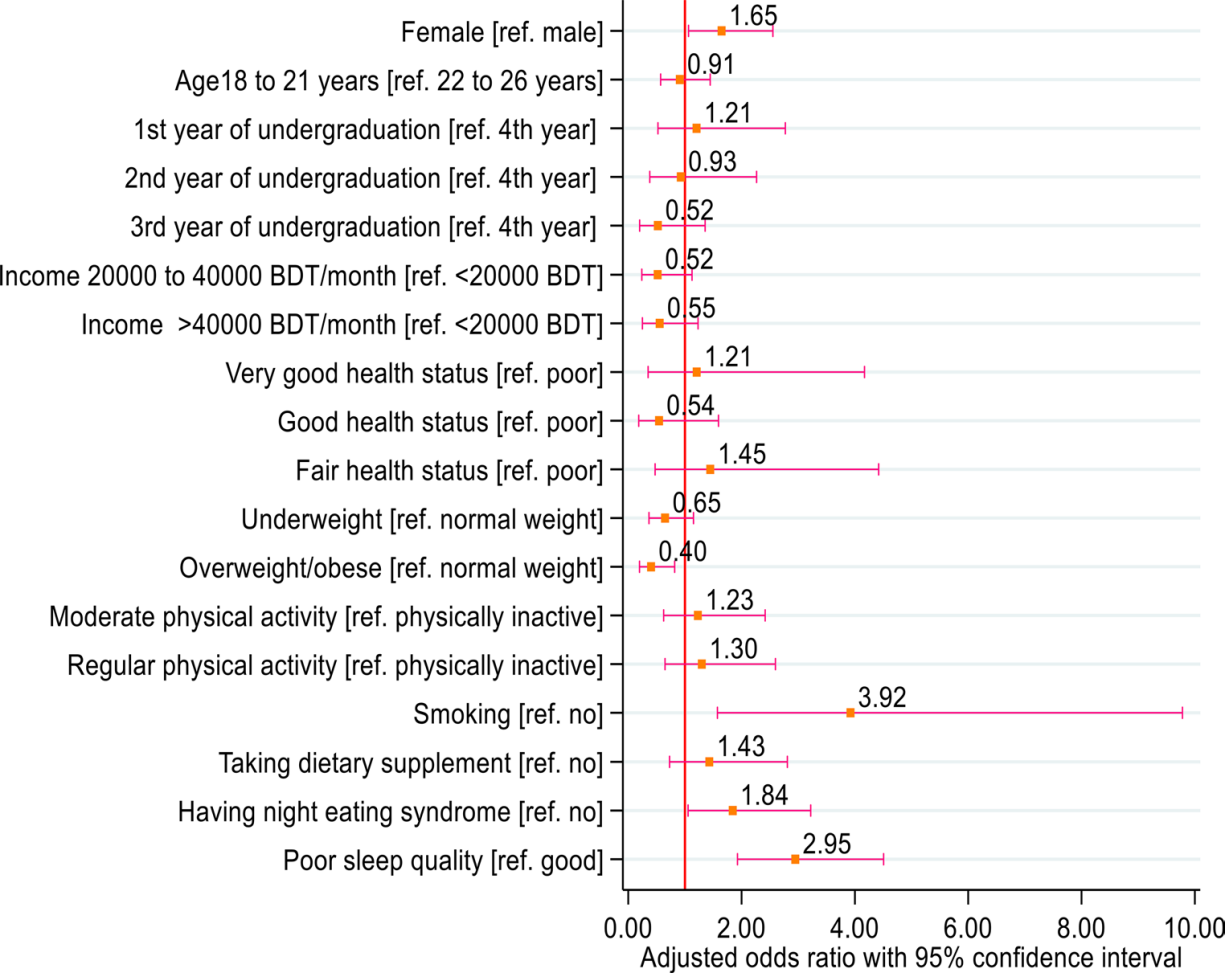
Odds ratio plot demonstrates the likelihood of skipping breakfast among study participants by different predictors. In each row, the square marker (orange color) and horizontal capped spike (pink color) range indicate the odds ratio and 95% confidence interval for a single variable. Statistical significance was ascertained when the corresponding 95% confidence interval did not exceed the red-colored reference border (odds ratio = 1)

The adjusted estimated effects of factors associated with skipping breakfast were shown in Table 4. The adjusted binary logistic regression analysis showed that female participants were more likely to skip breakfast compared to male participants (adjusted odds ratio, AOR = 1.65, 95% CI: 1.06 to 2.55, *p* = 0.031). Overweight/obese participants were less likely to skip breakfast compared to their counterparts (AOR = 0.40, 95% CI: 0.20 to 0.82, *p* = 0.012). Smoker participants had a higher likelihood of skipping breakfast compared to non-smokers (AOR = 3.92, 95% CI: 1.57 to 9.78, *p* = 0.003). Participants with night eating syndrome had a higher likelihood of skipping breakfast compared to their counterparts (AOR = 1.84, 95% CI: 1.06 to 3.22, *p* = 0.031). Participants with poor sleep quality were three times more likely to skip breakfast than their counterparts (AOR = 2.95, 95% CI: 1.93 to 4.51, *p* < 0.001) (see Fig. 2).

**Table 4:** Adjusted binary logistic regression showing the factors associated with skipping breakfast among study participants

Variable(s)	Adjusted Regression Model			
	Odds ratio	95% confidence interval		P value
		Lower limit	Upper limit	
Gender				
Male	Reference			
Female	1.65	1.06	2.55	**0.025**
Age (in years)				
18–21	0.91	0.57	1.45	0.688
22–26	Reference			
Study level				
1st year	1.21	0.52	2.77	0.661
2nd year	0.93	0.38	2.26	0.867
3rd year	0.52	0.20	1.36	0.182
4th year	Reference			
Monthly family income (BDT)				
<20,000	Reference			
20,000–40,000	0.52	0.24	1.13	0.097
>40,000	0.55	0.25	1.23	0.147
Self-perceived health status				
Very good	1.21	0.35	4.17	0.765
Good	0.54	0.18	1.59	0.266
Fair	1.45	0.47	4.42	0.517
Poor	Reference			
Self-reported body mass index				
Underweight	0.65	0.37	1.15	0.139
Normal weight	Reference			
Overweight/obese	0.40	0.20	0.82	**0.012**
Physical activity level				
Physically inactive	Reference			
Moderate activity	1.23	0.62	2.42	0.552
Regular activity	1.30	0.65	2.60	0.461
Smoking status				
Yes	3.92	1.57	9.78	**0.003**
No	Reference			
Taking dietary supplements				
Yes	1.43	0.73	2.81	0.299
No	Reference			
Night eating syndrome				
Yes	1.84	1.06	3.22	**0.031**
No	Reference			
Overall sleep quality				
Good	Reference			
Poor	2.95	1.93	4.51	**< 0.001**

*Note* Bolded values indicate statistically significant (*p* < 0.05). The adjusted regression model was fitted by the Hosmer and Lemeshow Test [chi-square (df) = 9.711 (8), p value = 0.286]

## Discussion

The study investigated the prevalence of skipping breakfast among university students in Bangladesh and its association with sociodemographic characteristics, sleep quality, and night eating syndrome. Key findings from this study suggest that the university students who are females, smokers, who have night eating syndrome, and those who have poor sleep quality have higher odds of skipping breakfast compared to their counterpart males, non-smokers, those without night eating syndrome, and those with good sleep quality. Breakfast adds to the quality and quantity of the required human body’s daily dietary intake and skipping it could have a profound effect on adverse health outcomes for individuals, especially for young adults. Knowing the pertinent factors related to skipping breakfast can help inform tailored program planning, design, and implementation to help reduce the prevalence of skipping breakfast and the associated adverse health effects.

A high prevalence of skipping breakfast (63.5%) among university students was observed in this study. This aligns with a previous study of 4th year medical students in Bangladesh [13], and is at the higher end of a range of estimates reported by additional studies investigating young adults [10] and urban-living adults [12]. In comparison to other countries, the prevalence recorded in this study is higher than 56.1% reported among university students in Malaysia [23], 55.4% reported among final year university medical students in Sri Lanka [24] and 28.9% also among university medical students in China [25]. However, in India and Ghana, cross-sectional studies involving undergraduate university medical students reported a higher skipping breakfast prevalence of 75% [26], and 71.92% [27], respectively. The reason for disparities in prevalence reported in different studies even in Bangladesh could plausibly be because of disparities in methods employed in collecting data, defining the outcome and differences in sociodemographic and socio-cultural characteristics present in each study area. Reasons given in other studies for students skipping breakfast include; waking up late, sleeping late, stress, dislike toward food choices, and the desire to lose weight [28]. Collectively, the prevalence of skipping breakfast is high across countries, thus, health promotion strategies and campaigns targeting university students that focus on promoting the benefits of eating breakfast may be a strategy to help reduce the prevalence of skipping breakfast.

Congruent to the findings from previous studies among urban adults in Bangladesh [12], university professionals in Bangladesh [29] and among public sector medical institutes in Karachi, Pakistan [30], this study found that females were more likely to skip breakfast compared to their male counterparts. This finding is however not on par with findings from previous studies conducted among undergraduate medical students in Bangladesh [13] and among Inner Mongolia medical students in China [25]. The plausible reason for females having higher odds of skipping breakfast than males could be because females are more concerned about their body shape or body image and some may adopt skipping breakfast as a means to lose weight [13]. This finding is particularly worrying because it has been reported that breakfast skipping among young females is characterized by a significantly higher incidence of dysmenorrhea [31], higher incidence of irregular menstruation [32], dysmenorrhea, and ovarian and uterine dysfunction [33, 34]. Dysmenorrhea which is often a symptom of gynecological condition could lead to frequent class absenteeism among female university students in Bangladesh.

In contrast to previous studies in Bangladesh [12, 29] and across other countries [10, 35, 36], this study found that university students who self-reported their BMI as overweight/obese were less likely to skip breakfast. The disparity in findings to similar studies could be explained by participants self-reporting their BMI in the present study. Considering that many studies [10, 12, 29, 35, 36] have reported that people who are overweight/obese have higher odds of skipping breakfast, this study’s finding may provide basis for further scientific investigation to help understand the reasons behind this disparity in findings. Perhaps another plausible reason could be that some overweight/obese students may be suffering from binge eating and disordered eating attitudes. A meta-analysis of longitudinal studies (age ≥ 18 years) reported that skipping breakfast are linked to weight gain and the development of overweight and obesity [37]. Further longitudinal studies are recommended to better understand the relationship between BMI and skipping breakfast among Bangladeshi university students.

Another key finding of this study was students who smoke were almost four times more likely to skip breakfast compared to non-smokers. Our finding is in agreement with several previous studies in Bangladesh [1], Japan [38], and Australia [39], which have consistently showed the association of smoking with breakfast skipping. Individuals who smoke often have poor self-rated health, poor nutrition knowledge, and poor health awareness [38, 40]; and this could plausibly explain why the university students in this study were more likely to skip breakfast. Few studies also found that smoking bears a positive correlation with poor sleep quality [41, 42], and this association adds a layer of complexity to this study findings, prompting us to delve the potential causal and/or moderating roles of smoking and sleep quality in relation to breakfast skipping. However, this study primarily focused on individual associations of variables such as smoking and sleep quality on breakfast skipping, which represents a limitation that future research may seek to address.

Studies across the countries explored the correlation between sleep quality and breakfast skipping, shedding light on how the factors interplay in various population groups [43–46]. This study strengthens the evidence by showing that university students who reported poor sleep quality were three times more likely to skip breakfast than their counterparts. The critical aspect of this connect is that sleep is linked to circadian rhythms, hormone regulation, and overall metabolic health, which in turn affect dietary habits [43–45, 47]. Students are often stressed with academic works, irregular schedules, and cultural activities, disrupting their sleep patterns, leading to poor sleep quality among them [48, 49]. A growing body of randomized controlled trials highlighted the need for accessible interventions such as exercise training, educational programs, and virtual self-care models, in which university students were largely ignorant [50–52]. That said, the university students are in need of support with regular sleep, which regulates appetite and satiety hormone, such as leptin and ghrelin, the higher education establishments and relevant departments should focus on evidence-based programs to address the problem.

In our sample, skipping breakfast was higher among students with night eating syndrome, which emphasizes the significance of this correlation. Night eating syndrome, characterized by increased food intake at night, can disrupt appetite regulation and energy balance, and this disruption is compounded when a person also skips breakfast as it prolongs the overnight fasting period. The association between breakfast skipping and night eating syndrome has been explored and substantiated in previous studies [38, 53, 54]. The combination of skipping breakfast and engaging in night eating syndrome behaviors can place these individuals at an elevated risk of various health outcomes, including obesity, insulin resistance, and cardiovascular diseases [53–55]. Globally, Clinical Practice Guidelines for the treatment of individuals with obesity are common, and focusing on patients’ ability to follow treatment plans, the guidelines suggest preventative measures such as lifestyles modifications through behavioral change models [56]. Here, public health professionals have a role to play in promoting health surveillance and in designing targeted health promotional interventions for university students.

## Limitations

It should be noted that our study had a few limitations. Firstly, the data collected relied on self-reported information, which may introduce recall bias and social desirability bias. Secondly, our sample was limited to young adults in a specific geographic area, which may not be representative of the entire population of university students in Bangladesh. Thirdly, the analysis did not control for semester time (because we didn’t obtain the exact timing of collecting the data in relation to examination or pre-examination period), which may have influenced the study results. Finally, causation cannot be established due to the cross-sectional nature of the study design. Vital statistics regarding nutritional practices and health of students are notably scarce in South Asian countries, including Bangladesh; the establishment of databases in these countries is recommended for regular and accurate health measurements and policy review. Future primary research could benefit from larger, more diverse samples and the inclusion of additional variables such as dietary preferences and mealtime routines to obtain a more comprehensive understanding of breakfast-skipping behaviors in young adults of Bangladesh.

### Recommendations: Support and interventions for university students

i. Ensure that university students have access to a medically-trained doctor and a dietitian for consultation; investigate their sleep quality and eating behaviors focusing on identified risk factors in the study such as smoking habits, night eating syndrome, and poor sleep quality.
ii. Evaluate the effectiveness of such consultations with consideration for students’ circadian rhythm and overall metabolic health.
iii. Ensure that instructional checklists on eating behaviors and available support and access information to intervention are included in their orientation program.
iv. Implement evidence-based health promotion strategies and campaigns targeting university students to raise awareness about the benefits of eating breakfast. Design interventions specifically tailored to address the higher likelihood of breakfast skipping among females, considering factors such as body image concerns and food neophobia.
v. Implement accessible interventions, such as exercise training programs, online food shopping platforms, and virtual self-care models, for ongoing support for students.
vi. Develop credit-bearing, online educational modules on eating behavior and sleep hygiene for students. Modules can be replicated by other universities by students and teachers.
vii. Further longitudinal students are required to (a) better understand the relationship between BMI and skipping breakfast among university students, and (b) investigate the potential influence of disordered eating behaviors among overweight/obese students as a contributing factor to their likelihood of skipping breakfast.
viii. Collaborate with higher education establishments locally, nationally, and internationally to raise awareness around students’ poor sleep quality, including academic stress and irregular schedules.

## Conclusion

This study revealed a high prevalence of skipping breakfast among a large sample of university students from Bangladesh. Significant associations were observed between skipping breakfast and gender, smoking behavior, night eating syndrome and sleep quality. Specifically, female students, smokers, those with night eating syndrome, and individuals experiencing poor sleep quality were more likely to skip breakfast. The observed prevalence of breakfast skipping surpasses estimates from various international studies, emphasizing the urgency of addressing this pervasive health behavior. Disparities in prevalence across countries, even within Bangladesh, underscore the need for tailored interventions considering sociocultural nuances. The unexpected inverse relationship between self-reported overweight/obesity and breakfast skipping warrants further exploration, possibly implicating disordered eating attitudes. The study also underscores the substantial influence of smoking on breakfast skipping, introducing a complex interplay with poor sleep quality. Accessible support systems, evidence-based interventions and further longitudinal studies are imperative to mitigate the adverse health effects associated with breakfast skipping among university students in Bangladesh and potentially beyond.

## Data Availability

The datasets used and/or analyzed during the current study are available from the corresponding author on reasonable request.
